# Extracts and Abstracts

**Published:** 1889-04

**Authors:** 


### EXTRACTS AND ABSTRACTS.

**Notes on the Value of Some New Drugs.** —The drugs that I purpose to say a word or two about to-night are paraldehyde, urethan, antipyrin, and pilocarpin; and perhaps, in dealing with antipyrin, I may say a word or two upon my experience with other antipyretic drugs. In these go-ahead days I may be taken to task for calling these new drugs, and I had some doubt how to name my paper; but if I cannot call them new I most certainly cannot call them old, and no equivalent to the "subacute" of medicine came ready to my hand. Let me also say that I do not propose to treat of these drugs from any other than my own standpoint; it is simply my object to say in what I have found them useful, and ask you for such experience as you may have of the same agents in similar or other fields. Thus I shall hardly, if at all, discuss the actions of the remedies, nor is it my object to give even a summary of the diseases in which they have been used or recommended.

Now, first of all, *paraldehyde.* It has been *out*, so to speak, for several years, but I do not think it is at all in common use. I am rather fond of it, and have given it frequently now certainly for three or four years, and that means in a large number of cases. Originally recommended, I believe, as a hypnotic, one that combined the advantages, to the exclusion of the disadvantages, of chloral and bromide of potassium, I tried it first of all in cases of insomnia. I do not consider it at all a reliable drug for that state of things; but it is a drug of unquestionable value in the restlessness and cardiac asthma so often seen in aortic disease, and in the laboring dilated heart of chronic Bright's disease. I well remember the first case in which I gave it some years ago, a man who had long been subject to gout, and who had a granular kidney and a large dilated heart. His distress was extreme, and I had exhausted all the old-fashioned remedies (than which few new are better), and many new, including caffeine, and with a marked condition of Cheyne-Stokes' breathing, he had all the aspect of speedy dissolution. As a last resource I gave him half-drachm doses of paraldehyde, with marked relief to the end of the case, which occurred about a fortnight later. I have also found it occasionally useful in the headache of renal disease. In two cases, where this was especially troublesome in the early morning, it was taken on and off for many months, with great relief. As a general rule, nitro-glycerine is the more reliable remedy for the headache of Bright's disease, but the symptom is often a troublesome one, and paraldehyde may well be kept in memory for occasional cases. But there is another class of cases in which I think it of even more use. It was recommended some few years ago, I think by Dr. Strachan, for certain cases of mania, and some three years or so ago I had to deal with a very troublesome case of alcoholic delirium. The patient was a very free drinker, had had delirium tremens twice before, and was now in a low, muttering, sleepless condition, which nothing seemed to quiet or control. He had been in a semi-comatose state already for five days. He was quite unheedful of any one about him, talked in an unintelligible mutter, and his muscles were tremulous, flabby, and wasted. As his wife was his equal, if not his better half, in drinking capacity, he was sent into Guy's Hospital, and for eighteen days he remained in much the same state. During that time his treatment consisted of free aperients, iodide and bromide of potassium, opium, and latterly of subcutaneous injections, first of $\frac{1}{100}$ grain, and then of $\frac{1}{60}$ grain of hyoscyamine; none of them seemed to do him any good. I then tried half a drachm of paraldehyde three times a day, and within a day or two he was much clearer in his mental condition, and before a week was out he was practically well. He remained somewhat feeble-minded for some little time, but he quite recovered eventually. Since then I have given it in similar cases several times. Whether it acts as a stimulant or a sedative I do not know, but it certainly sometimes quiets the delirium and clears the mental faculties. My friend Mr. Wornum lately ordered it in another such case, but it was only given in ten-minim doses. The general condition improved decidedly, but the mental condition still remains cloudy. However, the patient has come out of a semi-comatose state, and is now sitting up, although she still suffers from delusions.

Another case of what I think may be called a similar ailment was treated in a similar manner. A young fellow, aged 27, had typhoid fever badly; he became excessively emaciated, and not long after the fever left him he went off his head; he was very delirious and troublesome. Free stimulation failed to restore him, and he was, therefore, ordered half-drachm doses of paraldehyde. This was on the 8th of the month. The stuff was so nasty, he said, that he would take no more, and when I again saw him, on the 11th, it had not been persevered with. He then began it regularly, and within four or five days he was much clearer and less delirious, and at the end of a week he was practically well. * * * * *

Of *urethan* I have not much to say, but that little is in its favor. I have used it in a large number of cases as a hypnotic, and although it often fails, I think it is one of the best remedies of the kind since the introduction of chloral. I give it in fifteen-grain doses in water, and have often found it useful; and once I remember it was very so in relieving the headache of early typhoid.

Of *pilocarpin* I have only two points that I wish to make. I have, of course, used it quite commonly in Bright's disease, and still do as a usual thing, and it seldom fails to induce a copious diaphoresis; but one day it occurred to me to order a third of a grain as a subcutaneous injection in a case of chronic jaundice with intense itching. It occurred to me that a drug which so uniformly was productive of speedy diaphoresis must profoundly modify the functions of the skin for the time being, and might in doing so relieve the itching which has hitherto, in my experience, defied treatment. It did so quite beyond my expectation, and kept the patient comfortable until she died. In the next case it was equally successful. The patient had one-third of a grain injected subcutaneously many times, and always with this result, that the first twenty-four hours he was quite free, the second he was fairly free, and the third day he was getting bad again, and the dose had to be repeated. I next tried it on an old lady who suffers from very frequent attacks of gall-stones. She is equally decided as to the relief she obtains, but her pleasure is to some extent marred by her skin being for the time converted, as she describes it, into the similitude of that of a laundrywoman. I have had six cases in all, and in none has it failed; and when we consider that really there is nothing that can be relied upon to relieve this most distressing feature of jaundice, I hope the suggestion may prove of service. The other class of cases in which it seems to me to be occasionally of value is where the lightning pains of locomotor artaxy are severe. I have two or three times found it useful in these cases when many other means had been tried and failed.

Of *antipyrin* I am almost afraid to speak, for just now it is such a fashionable drug that it would well-nigh be easier to say for what diseases it had *not* been recommended than to enumerate those for which it had. But it does seem that it is likely to achieve a permanent name for abnormal nerve discharges of various kinds, and chiefly those that have been, I think happily, designated as the "paroxysmal neuroses." For instance, there can, I think, be no doubt that it is a very valuable means of combating some migrainous headaches. From what others have told me, and from what I have occasionally seen myself, it would appear to be a remedy of value in some cases of dysmenorrhœa—cases which might, I think, not unfairly be designated as pelvic catamenial neuralgia, if the gynæcologists will forgive me for venturing to have an idea outside my own lines. * * *

Antipyretics have been advocated by some in enthusiastic terms for the sudden and sharp febrile states that are met with in childhood, and I am far from saying that in such they may not be of use. I believe they may be of great use in that special condition known as hyperpyrexia —or rather in the states which threaten it —because in it the rise is very sudden, the attack is of the nature of a sudden storm-blast, and the tissues may be irreparably withered by it. In the same way in children sudden fever may do irreparable damage to their succulent tissues, and a dose of antifebrin or antipyrin may avert this. Nevertheless, it will occur to all of us that the attacks in which they seem to be so beneficial are just those whose nature is to subside as suddenly as they come, and I do not think that the evidence they afford is as yet conclusive in favor of these remedies.

But to go back to antipyrin in its power to depress abnormally high temperatures and to dissipate a migraine or pelvic neuralgia, we have evidence of some powerful influence on the nervous centers, and it is not far thence to the question, Might it be useful in other neurotic states? And it has been tried of late in two very troublesome ailments, namely, chorea and whooping-cough. I have no personal experience to relate as regards the latter, but it has been spoken of very favorably by Sonnenberger, Wendt, and Genser. Sonnenberger has tried it in about seventy cases, the dose varying from one-seventh of a grain in very young children to fifteen grains in adults three times a day. Wendt followed the former writer's suggestions, and states that the drug served him better than any other; he claims no cures, but it favors an easy course to final recovery, a mitigation of the paroxysms and a reduction in their number, and certainly a freedom from complications. Genser also asserts that antipyrin always diminishes the number of the coughs in the twenty-four hours, and controls the intensity; the duration of the disease seldom extended over twenty-four days—a very good record, as all will allow, if it be borne out by other observers. The mean dose was a grain and a half a day for each year of age; but I should say myself that the drug might be pushed to the extent of $1\frac{1}{2}$ grain per year three or four times in the twenty-four hours if necessary.

As regards chorea, I can speak—to some extent and tentatively—favorably. I have given it in the last five cases that have come under my observation. In four the result may, I think, be considered to have been good. Thus a girl, aged 8, was admitted for her fourth attack of chorea. It was general and rather severe, and she had mitral disease (probably contraction) also. She was put upon two-grain doses of antipyrin three times a day, and after five days it was noticed that there was a marked improvement in the choreic condition, the face muscles especially being quieter. The dose of the drug was then increased to four grains three times a day. She continued to improve for ten days, and by that time the movements had lost all their violence, when the drug was discontinued for arsenic. The minor movements remained for some time, as is usually the case with any treatment. She left the hospital practically well after seven weeks.

In another case a boy of 12, who had had rheumatic fever twice and chorea on and off for a long time, was admitted to Guy's Hospital with marked but not violent chorea of face and extremities and aortic regurgitation. He was put upon five-grain doses of the drug three times a day on the 24th of the month; on the 29th the movements were noticed to be less marked, and on the 31st they were not noticeable, and he remained well afterwards. He took the drug for twelve days altogether, and it was then discontinued because of an erythematous eruption that appeared. He was in the hospital three weeks.

A third case, a girl of 8, under the care of Dr. Cooper, of Rotherhithe—a very bad case indeed, associated with much wasting, imbecility, paralysis, and pericarditis—was given also five grains three times a day. She took it for eight days, and was by that time so much improved that it was discontinued for iron and digitalis. The improvement began within forty-eight hours after the commencement of the antipyrin.

A fourth case, now in Evelina Hospital, a girl of $9\frac{1}{2}$, came in on November 26th with general rather violent chorea. I gave her five-grain doses every six hours, and on the 29th increased it to ten grains every six hours. On December 3d she was lying in bed hardly choreic. In this case, however, although the chorea was manifestly better, she suffered from antipyrin poisoning. After three or four days a copious measly rash appeared, with high fever and albuminous urine, and then it was necessary to stop the drug.

The other case, under the care of Dr. Steele Perkins, of Streatham, was made worse by the treatment, and it was given up after ten days. I shall not indulge in any exuberant anticipations, but, remembering how very troublesome chorea is, I think there is enough evidence at hand to make us believe that, at any rate in some cases, antipyrin may prove of service. Legroux and Dupré have given it in twenty cases in doses of about seven grains five or six times a day, and they conclude that it is one of the most certain and harmless remedies for chorea, the improvement usually commencing in four to six days.

Before closing, perhaps I may be allowed to add a word not upon a new remedy, but upon an uncommon method of administration of an old one. I refer to the subcutaneous injection of *strychnine*. There is, of course, nothing novel in this. Nevertheless, I am under the impression that strychnine is not often administered in this fashion. Dr. Habershon has advocated its use thus in cases of cardiac failure, and during the past year I have had three troublesome cases of more or less general paralysis that have, for such cases, rapidly improved by its means. One was a case of peripheral neuritis due to alcohol and lead poisoning combined. He was in a very tremulous condition when admitted. Many of his muscles were much wasted. He had a good deal of pain in the course of various nerves, and he was both sleepless and demented. Various drugs were administered, and he was assiduously galvanized, but he became decidedly more powerless. By-the-bye he had a subcutaneous injection of hydriodate of hyoscine, first $\frac{1}{200}$ of a grain and then $\frac{1}{100}$, and this certainly seemed to quiet his nocturnal delirium, and procure him sleep. On November 4th I ordered him $\frac{1}{60}$ grain of strychnine to be injected hypodermically twice daily. On the 14th it was increased to $\frac{1}{50}$, and by the 31st he was taking $\frac{1}{34}$ twice daily. By this time it was noted that the movements of the patient's hands and arms were much more lively, and there was a gain in the grasping power of both hands. From this time he made continuous improvement. The injection was continued for another month, and was then omitted, chiefly because it caused, or he thought it did, severe pain in his ulnar nerves, which, he insisted, came on soon after the injection, and caused him great misery for several hours afterwards. He had had this pain before the commencement of the injections, and my clerk, Mr. McIlwaine, had doubts about the reality of the cause, but the man was very positive, and I think he was very probably correct. His ulnar nerves were clearly those most affected by his disease, and it is fair, therefore, to suppose that the remedy acted upon them in excess.

A second case was one of general paralysis, dysphagia, diaphragmatic palsy, and reaction of degeneration in the muscles in a young man aged 27. All the history that he could give was that, being a carman, and of late carrying sacks of coal, he had, a month before his admission, felt generally out of sorts and easily fatigued. A week later his finger-tips became numb, and then his toes, and gradually he lost all power in his legs, and then in his arms. He was at first given iodide and perchloride of mercury, and he steadily got worse. Then he had three-minim doses of liq. strychniæ given internally; but, as he was steadily going down hill, and was swallowing and breathing so badly that I quite feared for his life, I changed the internal administration of strychnine to the hypodermic method after a week. He had $\frac{1}{50}$ grain injected night and morning. This was commenced on the fifth, and on the ninth the report stated that there was a marked improvement in the strength of the right arm, and there was considerable improvement in sensation. By the thirteenth he was beginning to have slight control over his legs. His pains, before rather troublesome, were not so severe, and the patient seemed altogether better; and he rapidly improved from that time, so that at the end of a month he was able to get up and about feebly. He is now, many months later, quite well. It might be added that the real nature of the case still remains obscure to me. The completeness of the paralysis, coupled with his occupation, and a doubtful thickening and prominence over the second cervical vertebra, made me think at first of some cervical pachymeningitis, but I subsequently inclined more to the diagnosis of peripheral neuritis, albeit the cause of it could not even be suggested.

I must only inflict one other case upon you. It is that of a man aged 41, who was admitted for loss of power in his right leg, and incontinence of urine. He came in with these symptoms, and there was, on careful testing, some loss of sensation in the opposite leg. His bladder was running over. This and the paralysis made one suppose that there was disease in the cord, rather than outside it, and I feared he had some transverse myelitis, worse on the right side. He had not been in the hospital long when he lost the use of the other leg. He had at first some iodide of potassium and perchloride of mercury. But not finding any marked improvement after a fortnight, I changed it to two-minim doses of the liquor strychniæ of the *British Pharmacopœia*, twice a day, and at the end of five weeks he was up and walking about the ward. He is getting about fairly well now, with some clonus and exaggerated reflexes on both sides; but his chief trouble is frequent micturition, possibly due to some slight vesical irritation still remaining.—*James F. Goodhart, M.D., in the British Medical Journal.*

---

***The Relative Value of Opium, Morphine, and Codeine in Diabetes Mellitus.***—Since Pavy's recommendation of codeine as a remedy having advantages over opium and morphine in the treatment of diabetes mellitus, codeine has been much used, and has even to a large extent displaced opium and morphine in the treatment of this disease.

There are at the same time no clear pharmacological data in support of this preference. Indeed, the data are not even such as to suggest it; for notwithstanding assertions of a like superiority in the relief of various symptoms of other diseases, the facts, so far as we know them, seem to show that, pharmacologically, codeine is in its most important actions merely a weak morphine. This conclusion is supported by the circumstance that in its chemical structure it differs from morphine only in containing methyl—that in fact it is morphine diluted by the addition to it of the relatively inactive substance, methyl. In regard to its therapeutic effects in those conditions, other than diabetes, in which it is now commonly used, I have also failed to obtain any evidence that it acts otherwise than a weak morphine; for example, in the relief of cough, the production of sleep, or the removal of pain whether in the abdomen or elsewhere.

These considerations, as well as the special suitableness of the disease for such comparison, and the probable value to therapeutics of any decisive results that might be obtained, induced me, several years ago, to make some observations for the purpose of comparing the effects of codeine with those of opium and morphine upon the more important of the phenomena of diabetes mellitus.

Observations of a detailed character have been made on four patients, with results of a concordant kind; but I propose to describe, by way of illustration, the results that are of most importance in one of these patients only, on whom, incidentally, some observations were also made with atropine.

This patient, M. B., aged 26, came under my care in the Royal Infirmary of Edinburgh, on October 5th, 1887, suffering from thirst and frequent micturition. None of her immediate relations had suffered in a like manner. She was a nursery maid, and had lived in comfortable houses, and been supplied with abundant and good food. The first symptom of her illness was thirst, which showed itself about seventeen months prior to her admission, and had led to the discovery that she was suffering from diabetes mellitus. She was treated for this illness, but without much effect, and she soon became very weak, and lost flesh, so that in twelve months her weight was only eight stone, whereas immediately before this illness it had been ten stone. Notwithstanding careful and strict dieting, the emaciation advanced, and she weighed only seven stone four pounds when she entered the Royal Infirmary. She was then dyspeptic, and moderately anæmic, but she did not suffer from constipation, nor was the liver enlarged. There was no disease of the respiratory or circulatory systems. The eyes were hypermetropic, the skin dry, and the temperature slightly subnormal.

The patient was in the hospital from October 6th, 1887, to July 10th, 1888, and the observations to which I shall now draw attention occupied the whole of this time. The general plan followed was to allow the patient ordinary diet for one period; then to adopt a carefully restricted diet; then this diet with codeine, in doses varying from half a grain to five grains thrice daily, and for a short time with one-sixtieth and one-thirtieth of a grain of sulphate of atropine thrice daily; then restricted diet alone; then restricted diet, first with opium alone, in doses of half a grain to one grain and a half, and afterwards with opium and sulphate of atropine; and, finally, restricted diet with hydrochlorate of morphine. The administration of morphine was not preceded by an interval in which restricted diet alone, and without any medicinal treatment, was adopted; as in the period immediately preceding that of the administration of morphine, in which no medicine was given, the patient had become dull, languid and sleepless, she had complained of sickness and loss of appetite, and in a few days had been unable to leave her bed.

During the whole of the nine months in which the patient was under observation and treatment, with the exception of only a few days occurring now and then, the quantity of fluid drunk, the quantity of urine voided, and the specific gravity of the urine and the amount of urea and of sugar contained in it, were each day recorded. The weight of the patient was ascertained from time to time, and a careful record was made of her diet, and also of her general state of health. The daily observations are too numerous to be communicated to the Section.

To state the results in another way:

1. The case was one in which mere restriction of diet did not have so marked an effect as occurs in many cases. The prospects of successful treatment were not, therefore, very hopeful.

2. Codeine had a very decided effect in reducing the quantity of urine, sugar, and urea. When contrasted with the reduction produced by restricted dietary alone, the addition of nine grains of codeine in the day lessened by about one-third, and of fifteen grains of codeine in the day by about one-half, the quantity of fluids drunk, and the quantity of urine, sugar, and urea, and it slightly reduced the specific gravity of the urine.

3. The addition to fifteen grains of codeine of the one-twentieth, and afterwards of the one-tenth, of a grain of sulphate of atropine, caused a still further, though not a large, reduction.

4. After the administration of codeine had been stopped, an interval of six days on restricted diet, without any medicinal treatment, was not sufficient for a deterioration to occur to the conditions present before codeine had been given.

5. The subsequent administration of half a grain of opium thrice daily produced a considerable reduction. With one grain of opium thrice daily the reduction was to less than one-half, when contrasted with the amounts during a restricted diet alone, and before any medicinal treatment had been adopted. One grain and a half thrice daily produced a further reduction; and when to it was added one-twentieth of a grain daily of sulphate of atropine, a still further reduction occurred.

6. Restricted diet, with one-third of a grain of hydrochlorate of morphine thrice daily, or one grain daily, also produced a marked reduction; and the conditions relative to the points under investigation were even more satisfactory than when fifteen grains daily of codeine were being administered. While this small quantity of morphine was being taken, the fluids drunk by the patient were only one-third, the urine and sugar less than one-half, and the urea about one-half of the amounts during the period of restricted diet alone, before medicinal treatment had been commenced.

As to the general state of the patient during each of these conditions of treatment, restriction to an antidiabetic dietary produced improvement in thirst and mental activity. So long as the quantity of codeine was limited to six grains daily, this improvement was maintained; but when nine grains, and even more, when fifteen grains were being taken daily, the appetite failed, she became listless and apathetic, vertigo was occasionally experienced, and the patient remained for a considerable part of each day asleep in bed. The addition of atropine to the codeine did not produce any improvement, but rather added to the discomfort by impairing vision; and even when only one-sixtieth of a grain was being taken thrice daily, the pupils became slightly dilated. When codeine and atropine were stopped, and a restricted dietary alone adopted, the health was not improved. The symptoms referred to became, indeed, worse, and prevented a prolongation of the period of restricted diet without medicine to the extent that seemed desirable before a new plan of treatment was adopted. When opium was now given a marked improvement occurred, but the larger doses caused some drowsiness during the day. With one grain daily of hydrochlorate of morphine the condition of the patient became more satisfactory. The drowsiness soon disappeared, the appetite improved, and she became sufficiently active to engage in ward work. Constipation of the bowels was not produced by any of the medicinal agents employed.

A consideration of these averages seems to show that, under a daily administration of one grain of hydrochlorate of morphine, the quantity of fluids drunk, and of urine, urea, and sugar voided, was rather less than when three grains of opium, and decidedly less than when fifteen grains of codeine were being taken. In three other cases in which I have instituted a comparison between these substances in diabetes mellitus, morphine also showed a marked, though not so great, superiority over codeine. After this note had been prepared, I have seen a recent paper by Dr. Bruce, of London, in which similar results were obtained in two very carefully observed cases. So far as I know, also, the favor with which codeine is regarded in this disease has not been supported by any observations calculated to show its value relatively to opium or morphine so clearly as in the cases to which I have referred.

The evidence, therefore, seems to indicate that codeine is a less powerful remedy in diabetes than either opium or morphine, and to confirm the view that in its therapeutic value it ranks as a weak or diluted morphine.

The conclusion receives an importance (no doubt a subsidiary one) from the circumstance that codeine is about three times as expensive a substance as morphine. The great demand for it has led to its being manufactured from morphine so largely, that probably one-fourth of the codeine in the market is an artificial substance. When we consider the large doses that are required in diabetes mellitus, and the generally protracted duration of this disease, we are, I think, justified in asking for more clear evidence of its superiority over morphine than has as yet been produced.—*Thomas R. Fraser, M.D., in The British Medical Journal.*

---

***Guy's Hospital Lying-in Charity.***—In volume XLV of *Guy's Hospital Reports*, recently issued, may be found the sixth report of the lying-in charity of the Hospital, from October 1, 1875, to September 30, 1885, collated from the records by Dr. P. Horrocks, and published by the collator and Dr. Gallabin. The report is interesting on account of the number of cases confined in the Hospital during the ten years —25,489, or an average of 2,548 a year.

The analysis of births is as follows: 25,489 women attended, with 25,777 births; single births 25,201; dual births 558, triple births 18. The viable children living at birth were 24,640, or 96.46 per centum; viable children stillborn 983, or 3.84 per centum; undescribed 13—total 25,636. Non-viable children living (few minutes to several hours) 17; non-viable children stillborn 124. Of the viable children 13,520 were males; and 12,097 females, the ratio being as 100 to 89. In 19 the sex was undescribed. Of the non-viable children 56 were males, 34 females, and 51 undescribed.

Of the 24,582 viable living children of whom the sex is given, 12,941 were males and 11,641 females, or as 100 to 90.

The presentations were as follows:

| Presentation. | Males. | Females. | Total. | Per cent. |
|---|---|---|---|---|
| Vertex | 12,622 | 11,316 | 23,938 | 97.38 |
| " and head | 36 | 20 | 56 | .23 |
| Breech | 153 | 178 | 331 | 1.34 |
| Foot | 60 | 72 | 132 | .54 |
| Face | 41 | 32 | 73 | .30 |
| Upper extremity | 7 | 5 | 12 | .05 |
| Transverse | 2 | 7 | 9 | .04 |
| Brow | 8 | 4 | 12 | .05 |
| Funis | 12 | 7 | 19 | .07 |

Of the viable stillborn children 547 were males and 425 females, or as 100 to 78. The presentations were as follows:

| Presentation. | Condition. | Males. | | Condition. | Females. | | Total. | Per cent. |
|---|---|---|---|---|---|---|---|---|
| Vertex | Full term | 326 | 367 | Full term | 246 | 285 | 652 | = 67·1 |
| | Decomposed | 3 | | Decomposed | 6 | | | |
| | Premature | 38 | | Premature | 33 | | | |
| Vertex and hand | Full term | 5 | 6 | Full term | 4 | 5 | 11 | = 1·1 |
| | Premature | 1 | | Premature | 1 | | | |
| Breech | Full term | 57 | 66 | Full term | 62 | 67 | 133 | = 13.7 |
| | Decomposed | 1 | | Decomposed | 2 | | | |
| | Premature | 8 | | Premature | 3 | | | |
| Foot | Full term | 44 | 58 | Full term | 30 | 34 | 92 | = 9·5 |
| | Decomposed | 1 | | Decomposed | 0 | | | |
| | Premature | 13 | | Premature | 4 | | | |
| Face | | | 4 | | | 3 | 7 | = ·7 |
| Upper extremity | Full term | 15 | 16 | Full term | 7 | 7 | 23 | = 2·4 |
| | Premature | 1 | | Premature | 0 | | | |
| Transverse | | | 7 | | | 1 | 8 | = ·8 |
| Brow | | | 2 | | | 0 | 2 | = ·2 |
| Funis | With vertex | 12 | 21 | With vertex | 12 | 23 | 44 | = 4·5 |
| | " breech | 2 | | " breech | 1 | | | |
| | " foot | 1 | | " foot | 6 | | | |
| | " arm | 5 | | " arm | 3 | | | |
| | " transverse | 1 | | " transverse | 1 | | | |
| | | | 547 | | | 425 | 972 | |

There does not appear to be any relation between the sex of the children and the fact of the amniotic cavity or placenta being double or single.

There were, of the 6 cases of triplets, all males in 1 case, all females in 3, one male and two females, and two males and one female, 1 each. The presentations were:

Vertex, vertex and vertex..........2
" " " breech..........1
" breech " foot............2
" " " transverse.......1

Out of 18 children, 4 were stillborn, a mortality of 22.2 per centum, or 1 in 4.5. No instrumental aid was given in any of these cases. In one case the woman was a primipara.

There were 80 face presentations, giving 73 living and 7 dead children. In 74 cases delivery was accomplished by natural efforts, 5 children dying, one being an anencephalous fœtus. In 6 cases instruments were used, the forceps in 4, the cephalotribe in 2. In the forceps cases all the children were born alive, though one died within twenty-four hours. In two cases the forceps were removed after some traction had been made, and the cases were left to nature. Excluding the two anencephalous fœtuses, there were 5 deaths in 78 cases of face presentation, or 6.4 per centum.

The following table shows the proportionate number of the several presentations for the whole number of viable children, and the percentage of such children stillborn under each presentation:

| Presentation. | | | Per cent. | Percentage stillborn. |
|---|---|---|---|---|
| Vertex | 24,590 | = | 96·22 | 2·7 |
| Vertex and hand | 67 | = | ·26 | 16·4 |
| Breech | 464 | = | 1·82 | 28·7 |
| Foot or knee | 224 | = | ·88 | 41·1 |
| Face | 80 | = | ·31 | 8·7 |
| Upper extremity | 35 | = | ·14 | 65·7 |
| Transverse | 17 | = | ·07 | 47·0 |
| Brow | 14 | = | ·05 | 14·3 |
| Funis | 63 | = | ·25 | 69·8 |
| | 25,554 | | 100·00 | |

The death-rate in the viable children in the present report is 3·84 per cent. In the last report the death-rate in children was 4·08, in the last but one 4·6, and in the preceding twenty-one years it was 5·2 per cent. In connection with this great improvement, it is interesting to note the greatly increased use of the forceps. Not only have they been used twice as often as in the last series, but recourse has been had to their use at an earlier period of the delayed labor, and thus the children have had a better chance.

Taking foot and breech presentations together, the children stillborn are in the proportion of 1 in 3. In the last report the proportion was 1 in 2·5, and in the last but one 1 in 2.7. There were 279 twin cases, or about 1 in 91 of the whole number of women delivered, or about 1·09 per centum. In 89 cases both were males, both females in 88, and one of each in 102 cases. The presentations were:

| Presentations. | |
|---|---|
| Vertex in both | 125 |
| " and vertex with hand | 6 |
| " and breech | 72 |
| " and foot | 36 |
| " and upper extremity | 3 |
| " and transverse | 2 |
| " and face | 1 |
| Breech in both | 12 |
| " and foot | 7 |
| " and upper extremity | 1 |
| Foot in both | 8 |
| Not given | 6 |
| | 279 |

There were 63 cases of funis presentation, with 19 living and 44 dead children. In the last report only 8 were living out of 62. Of the present series there were 36 vertex presentations, 16 breech or foot, 9 upper extremity and 2 transverse. In 7 cases, of which 4 were vertex, 1 transverse, 1 upper extremity, and 1 breech, the funis had ceased to pulsate before the arrival of assistance. In 16 cases version was performed; 11 podalic, 2 cephalic, and 3 not stated. In one case of podalic version the child was alive; all the others were stillborn. Forceps were used in 8 cases, of which 6 were vertex and 2 upper extremity. In one of the vertex cases the child was saved; the others were dead. The funis was replaced in several cases, and in 4 cases it remained in the uterus. Of these 4 cases, 3 were vertex and 1 upper extremity, the last being stillborn, but in the 3 vertex cases the children were saved. Craniotomy was performed in 4 cases.

The brow presentations numbered 14, with 12 living and 2 dead children. Version was performed in two cases, both children dying; in one of these the forceps was used to the after-coming head. The forceps was used in 1 case, and the child born alive. Eleven cases were left to nature, all being born alive, the presentation being converted into a face in some cases. It appears that the child has a much better chance in brow presentations if left to nature or if forceps are used.

The upper extremity presented in 44 cases, and in 9 the funis was prolapsed. There were 19 transverse presentations, and in 2 the funis was prolapsed. Of the 63 cases, 6 were completed by natural efforts, 4 by spontaneous evolution, all stillborn, one by spontaneous version, and one case was delivered spontaneously, but by what method is not stated. Version was performed in 50 cases, 18 children being living and 32 stillborn. Podalic version was performed in 45 cases, all the mothers recovering save one. In the 3 cephalic version cases one child was living and the other two were dead. In 2 cases the kind of version is not stated. Of the 63 cases, 22 children were living and 41 were dead. Comparison with the last report shows that the number of cases of presentation of the upper extremity and transverse presentations has decreased, and that the mortality of the children is very much less. In the last report deaths of two mothers were recorded; in this only one.

Out of 25,636 births of viable children, protracted labor was terminated by forceps in 274, or 1 in 93—about 1.07 per centum. In the last report, out of 23,591 women attended, 121 protracted labors were terminated by forceps, or 1 in 97—0.51 per centum. In the present series, therefore, the forceps have been used more than twice as often as in the former series; but this report includes the cases in which forceps were used for accidental hæmorrhage and for eclampsia, which were omitted from the last report. The increased use of the forceps has undoubtedly saved many mothers a great deal of pain, reduced the mortality of the children, and probably also of the mothers; but it did not decrease the number of craniotomy cases, so that it will be seen that forceps have been used more frequently in cases of uterine inertia and slight obstruction, which might perhaps have been terminated by natural efforts alone if left long enough.

Of the 274 forceps cases, 11 mothers died; 6 from septicæmia, 3 from hæmorrhage, 1 from bronchitis, and 1 from heart disease; making the mortality 4 per centum, practically the same as in the last report. Of the 274 children, 237 were living, 36 dead, and 1 undescribed. Excluding the child about which there is no statement, we have 36 dead out of 273, a mortality of 13.18 per centum, or almost 1 in 7.6. Excluding one whose sex is undescribed and the one of which there is no information, we have 25 males dead out of 166 cases, a mortality of 15.06 per centum, or 1 in 6.6, while we have 11 females dead out of 106, a mortality of 10.37 per centum, or 1 in 9.6. In the last report the general infantile mortality was 24 per centum (nearly), the male mortality 30 per centum (nearly), and the female mortality 15 per centum (nearly). The increased use of the forceps in such cases does not add any dangers to the mother or child.

Forceps were used at the brim 92 times; 6 mothers and 18 children died. Version was performed when the head was at the brim in 33 cases, 5 mothers and 29 children dying, thus showing that the forceps give a much better chance to both mother and child than version. A tabular statement of all the forceps cases is given in the report, as well as details of specially interesting cases.

Version was performed in 90 out of the 25,636 viable cases, equal to 0.35 per centum, or nearly 1 in 285. Podalic version was the method chosen in 80 cases, cephalic in 3, and in 7 cases the record is imperfect. The version was performed for malpresentation in 49 cases, in most of which some part of the upper extremity presented. Of these cases 29 children (20 males and 9 females) were stillborn, and 20 (9 males and 11 females) were saved. Placenta prævia, with its accompanying hæmorrhage, was the cause of interference in 26 cases, of which 23 (14 males and 9 females) were stillborn, and 3 (2 males and 1 female) were saved. Version was performed for contracted pelvis in 14 cases, in most of which the conjugate diameter of the brim was chiefly or entirely at fault; in several the forceps were first tried in vain. Of the 14 cases, 11 (7 males and 4 females) were stillborn, and 3 (1 male and 2 females) were saved. In one case accidental hæmorrhage necessitated quick delivery, and version was performed. The child died. In 8 cases version followed by traction failed to deliver, and craniotomy of the after-coming head was necessary.

Of the 90 cases of version, 26 children (12 males and 14 females) were born alive, and 64 (41 males and 23 females) were stillborn. Excluding the 8 cases in which craniotomy was performed, there were 56 stillborn children in 82 cases of version—nearly 68.3 per centum, or 1 in 1.46. Of the mothers, 5, or 5.5 per centum, or 1 in 18, died; 4 were placenta prævia cases, and 1 was a case of contracted pelvis. Of the 5 deaths, puerperal septicæmia with peritonitis was the cause in 2 cases; puerperal septicæmia and hæmorrhage in 1 case; hæmorrhage in 1 case, and internal hæmorrhage (?) in 1 case. In all 5 cases the children were stillborn. Recently in placenta prævia cases, the forceps have been used instead of version. with greatly improved results to both mothers and children. In most cases the combined internal and external method, as described by Braxton Hicks, was adopted.

Craniotomy was performed 24 times, or 1 in 1,074, or in 0.09 per centum of the cases. In the last report the proportion was 1 in 1,310. In 7 of the 24 cases the children were dead before the operation was begun, in 15 cases life was a matter of doubt, and in but 2 cases was the child known to be alive. "At the same time, it must be remembered that in some cases the child's life was placed in fatal jeopardy by attempts at delivery prior to the use of the perforator." Four of the mothers died, one (two?) from rupture of the uterus, and two from suppurative peritonitis. The causes that led to the craniotomies were: narrow pelvis 16 cases, hydrocephalus 2, large child, atresia vaginæ, and tedious labor 1 each, not stated 3. In 9 cases the forceps were tried in vain; in 6 cases the forceps and then version were tried in vain; and in 2 cases version alone was tried.

There were 5 cases of rupture of the uterus or vagina, or 1 in 5,098. All the patients died, including 2 in whom laparotomy was performed.

The number of cases of post-partum hæmorrhage is 342, or 1 in 74, or 1.3 per centum; 14 mothers died, and 38 children were stillborn. Of the 14 maternal deaths, 7 were from hæmorrhage alone, 4 from subsequent septicæmia, 1 from scarlet fever, 1 from erysipelas, and 1 from heart disease. In 14 cases the hæmorrhage was arrested by injecting into the uterus a solution of perchloride of iron (1 part of the strong solution to 3 or 8 parts of water); 13 of these cases recovered. In the majority of cases ergot was given, a teaspoonful of the powder or of the liquid extract, the hæmorrhage ceasing very soon, except in a few cases. The use of ice in the vagina was unsatisfactory. In 35 cases the placenta was retained, and had to be removed by introducing the hand into the uterus; in 30 of these the placenta was adherent, and detached in 5. In 6 cases the mothers died. In 31 of the 96 reported cases more or less pyrexia, with accompanying febrile symptoms, followed; in 10 cases there had been antepartum hæmorrhage; in 10 cases version had been performed; in 33 cases the forceps had been used.

Placenta prævia occurred in 51 cases, or 0.2 per centum. Of the cases, 14 were complete, 22 partial, and 15 doubtful or not stated. There was more or less hæmorrhage in 50 cases; 9 mothers died, or 17.6 per centum—nearly 1 in 6; 4 from hæmorrhage, 4 from septicæmia, and 1 from rupture of the uterus; 34 children were stillborn, or 66 per centum. In 2 cases the patients died undelivered. Version was performed in 26 cases. "There can be no doubt that when a placenta prævia has been found, it is best to plug the cervix in order to stop further hæmorrhage. Barnes's bags answer well because they dilate the os at the same time. The case should be carefully watched, and a larger bag put in as soon as may be required. Then by rupturing the membranes, and, if necessary, separating the placenta from the lower zone, and delivering the child by forceps or version the risk of further loss of blood is diminished. If the vertex is presenting, the use of the forceps gives the child a better chance than version, but inasmuch as the labor is often premature, the ordinary forceps are apt to slip off."

There were 61 cases of puerperal fevers, or 1 in 418, or 0.24 per centum; 44 were fatal, making the mortality from this cause 1.7 in 1000. It is the rule to suspend an externe from his duties whenever one of his cases develops serious symptoms. In 1884, during the absence of the assistant obstetric physician, one of the externs was allowed to be a dresser in the surgery wards at the same time. No less than three of the women attended by him died of puerperal fever.

The principles of treatment are to remove any source of infection, wash out the uterus and vagina as necessary, and give a purge at the outset, with opium and hot fomentations. Quinine in 5 grain doses has been used as an antipyretic, and antiseptic measures are used.

In regard to maternal mortality, there were 86 deaths, or 3.4 per 1,000, or 1 in 296. In the last report the mortality was 4.4 per 1,000. Considering the poverty and destitution of most of the women, this further reduction of an already low mortality is very satisfactory.

---

***A Practical Point in the Treatment of Pott's Disease of the Spine.***—In a paper on this subject, read before the American Orthopædic Association at its annual meeting, September 18, 1888, Dr. A. B. Judson says: The treatment of Pott's disease is always open to amendment. I propose, therefore, in order to assist the practitioner, a simple rule, as follows: The efficiency of the apparatus used is to be measured by the condition of the skin covering the projection.

A few words in explanation will not be out of place. It is premised that the object of mechanical treatment is to make pressure on the projection with the threefold purpose of (1) enforcing fixation of the bones involved, (2) transferring pressure from the diseased bodies to the healthy processes, and (3) lessening the deformity. If we were dealing with the skeleton deprived of its integument there would practically be no limit to the pressure which might be applied to the projection. But the interposition between the apparatus and the vertebral column of the sensitive skin places a peremptory limit to the degree of force which it is possible to apply. The rule may therefore read: The apparatus may be considered as having reached the limit of its efficiency if it makes the greatest possible pressure on the projection compatible with the comfort and integrity of the skin.

This rule may be followed with perfect regard to the comfort and convenience and relief of the patient. If, contrary to common sense, the apparatus is fastened at once as tight as it can be borne, the skin will react speedily with pain and ulceration. But if the pressure is lightly applied at first, as may be conveniently done with apparatus constructed with this in view, and gradually and carefully increased from time to time, it will be found as the weeks and months go by that the skin has become indurated without losing its integrity or causing inconvenience, and its condition will be unimpeachable evidence that, so far as mechanical means go, the patient is receiving a full measure of the benefit of treatment.

Through the negligence of the nurse or the willfulness of the patient, apparatus designed for use in this way may produce, and occasionally has produced, abrasion and ulceration. But this should not be considered a good reason for condemning the apparatus. It is well to bear in mind that the disease in question is perhaps the most insidious in its progress and disastrous in its results of all the affections to which the young skeleton is liable. Shall we not make use of the most efficient method at hand and, if necessary, redouble our carefulness and painstaking in order to avoid incidental excoriation?

There are methods of treatment to which the rule proposed does not apply very closely. I refer to those methods which have for their principal features suspension and plastic dressings. If the patient is suspended and plaster-of-paris applied, the pressure is diffused rather than concentrated, and changes in the skin are less marked and it is less easy to adjust the direction and degree of pressure on given points, while it is especially easy and convenient to do this with a brace constructed of tractable steel. I would not decry the use of plastic dressings in the treatment of this affection. They are daily affording comfort and relief to numbers of sufferers who would otherwise have nothing done for them. They will continue, perhaps, to be used by the general surgical practitioner. But I think the specialist in orthopœdic practice can do better. He has a better method at his command in the use of tractable steel modified to meet the varying requirements of the case to which he devotes himself with the patience and ingenuity which are a part of his equipment.

To the practitioner of this class I commend the rule suggested above to the effect that the question of the efficiency of the apparatus is to be determined by the condition of the skin covering the projection, a rule which many of us have doubtless followed in practice, and which it can do no harm to have expressed in words.

---

*Cork as a Compress in Hæmorrhage.*—The surgeon frequently finds himself at a loss for a ready means of controlling certain hæmorrhages which, although seemingly insignificant at first, become quite serious in a very short time. This state of things is of course more likely to occur in the experience of the country surgeon than in that of his more fortunate city brother. In any event we are quite sure that the surgeon can find nothing so convenient and reliable for controlling hæmorrhages of the scalp, palmar arch, hands, feet, etc., and the oozing which often follows the too early or accidental removal of the remains of the umbilical cord, as the cork compress. The degree of resistance afforded by the cork is perhaps a little greater than that of one's finger, but the pressure secured by it is always uniform and reliable. The mode of its application is very simple. For scalp wounds a circular tap, about one quarter of an inch thick, is cut off the end of a cork about one inch in diameter, and secured to a bandage by a few drops of ordinary sealing wax. The section of cork thus prepared is placed over the bleeding vessel, and firmly secured by passing the bandage around the head in the usual manner as often as is necessary. For umbilical hæmorrhage the same description of cork and bandage is used, but the center of the cork is slightly scooped out for the accommodation of a small pledget of styptic cotton. By this means we have been able to control this very troublesome oozing when all other means known to us had failed. For wounds of the palmar arch the cork is cut in half, longitudinally, the flat surface secured to the bandage by a few drops of sealing wax, and the circular or rounded side pressed firmly along the course of the artery on the proximal side of the wound. The bandage is then passed firmly around the wrist often enough to secure the desired amount of pressure upon the wounded vessel. In the same way the compress is applied in wounds of the hands and feet. Patients never complain of any discomfort from the pressure thus applied.

Our experience with this simple method of compression prompts us to recommend it as a safe and efficient means of controlling superficial hæmorrhages of every description where ligation is unnecessary, inconvenient, or not desired.—*International Journal of Surgery*, March, 1889.

---

***Opening Deep Peri-Rectal Abscesses by Perineal Incision.***—H. Zeller (*Beiträge zur klinische Chirurgie*, Bd. iii., Hft. 2), referring to the abscesses above the pelvic diaphragm, states that these are usually found in the prostatic and peri-prostatic connective tissues. Abscess of the prostate itself is most frequently the result of irritation in the urethra; rarely by propagation of an ulceration from the rectum. Abscesses occurring primarily in the peri-prostatic connective tissue, and involving the prostate secondarily arise from extension of inflammation, or infection from neighboring organs. Further, ulceration of the bowel high up (stercoraceous abscess) may produce abscess in this locality. Abscesses originating in the vertebræ seldom find their way to this locality. Lastly, a series of cases is mentioned, in which the cause is not noted.

Rupture of an abscess purely prostatic in its origin occurs oftenest in the urethra, and at times is characterized by an intermittent discharge of pus; it very seldom breaks through into the bladder. Abscess of the peri-prostatic tissues open oftenest into the rectum; next in frequency into the urethra, and often into both rectum and urethra. They seldom point spontaneously towards the perineum, and are quite as likely to follow a direction towards the inguinal region, the ischio-rectal fossa, or the obturator foramen.

The prognosis of deep perineal abscesses is unfavorable. In 114 cases, 70 were cured, 7 terminated in fistulæ, while 34 died. The importance of early interference is insisted upon. The perineal incision recommended by Lallemand, even when no prominence in the perineum is observed, and when but slight arching toward the rectum can be felt, is recommended.

When decided bulging and fluctuation are observed in the rectum, there is a temptation to incise at this point. The fact that the rectal opening cannot be made sufficiently large for purposes of free drainage; that it is often not at the most dependent portion of the abscess; and further, the impossibility of antisepsis, and the risk of establishing a urethro-rectal abscess in the cases in which spontaneous opening into the urethra also occur;—these considerations together with the danger of hæmorrhage, will impel the surgeon to adopt the course recommended by the author.

---

***Tuberculosis of the Sacro-Iliac Joint.***—Dr. Weller Van Hook, in a paper on this subject, draws the following conclusions in regard to treatment:

1. Sacro-iliac disease is not directly amenable to treatment by drugs. They should, nevertheless, be employed by the surgeon in all forms of the disease when they are likely to improve the general condition of the patient.

2. Counter-irritation is indicated when there is pain, lameness or tumefaction at the joint without abscess formation. The actual cautery seems to be the most effective form.

3. Mechanical rest, which is here also physiological rest, is the treatment par excellence where no abscesses are present.

4. When abscesses have formed radical operative interference must be immediately resorted to.

5. If the abscesses are extra-pelvic they should be operated upon by direct incision and évidement.

6. When the abscesses are intra-pelvic the operator should approach the disease focus, supposedly in the anterior of the joint, by an opening made above the joint proper as described.

7. When both extra- and intra-pelvic abscesses are present the external abscess should be first opened, and the opening between the two, if possible, so enlarged as to admit of the radical treatment of the deeper focus of disease.

8. Radical operations cannot be made through long sinuses.

9. Drainage alone is not likely to be successful.

10. After-treatment should include besides antisepsis, continual rest, aided when necessary by the extension and pelvic belt.

11. When radical operation is undertaken no tubercular matter should be left behind.—*Annals of Surgery*, Feb., 1889.

---

***Benzoated Chloroform.***—Dr. B. W. Richardson recommends the use of benzoated chloroform as an antiseptic of considerable service in the treatment of fœtid wounds. It is made by dissolving three drachms of pure benzoic acid in twelve ounces of chloroform and filtering if necessary. In a case of fœtid ulcer of the lower extremities, after the bandage has been applied, he prescribes a fluid drachm of the solution poured over or near the ulcer, the deodorizing effect being of the best character. He states that the solution is also the most effective that he knows of for removing the fœtor in troublesome cases of fœtid exhalations of the feet. Used like eau de cologne, he finds it advantageous to rub over the hands at a post-mortem examination, and for similar purposes where a disinfectant is required.—*Asclepiad*, Vol. v., No. 19.

---

***Crude Sulpho-Carbolic Acid as a Means of Disinfection.***—E. Laplace (*Deutsche Med. Wochenschrift*, 1888, No. 7), following up his experiments which resulted in considerably increasing the antiseptic power of sublimate by the addition of acids, sought to obtain similar effects in carbolic acid. Realizing that the comparatively insoluble 25 per cent. crude carbolic acid is not available for disinfectant purposes, he added to the latter equal parts of crude sulphuric acid, heating the mixture and allowing it to cool. The result is a thick, black fluid, very easily soluble in water, which possesses undoubted antiseptic powers. For instance, it destroys the spores of anthrax, in a 4 per cent. solution, in forty-eight hours; a 2 per cent. solution will accomplish the same result in seventy-two hours. This is about equivalent to the performances of a 2 per cent. solution of pure carbolic acid. It would seem, therefore, that the principal advantage of this compound resides in its comparative cheapness.

---

***Radical Cure of Hypertrophy of the Prostate.***—Simultaneously with Casper, Dr. Roux, of Lausanne, employed the galvanic current in the treatment of hypertrophy of the prostate. He has used the method in six cases and claims to have obtained even better results than Casper. His procedure is as follows: A broad electrode is applied to the abdomen. The other, which consists of long needles varnished to within 12 to 15 millimeters of the point, is introduced into the prostate through the rectum. At the beginning of the application a weak current is employed, but in one to three minutes this is gradually increased, until the desired maximum is reached. Roux uses currents up to 70 milliamperes, which are applied for 2½ to 5 minutes in this strength, and then gradually diminished, while the needles are withdrawn and inserted into another part of the prostate. No especial disinfection is required, since Roux regards the galvanic current itself as a sufficient disinfectant.—*Deutsche Medizinal Zeitung*, No. 97, 1888.

*Beta-Napthol in Typhoid Fever.*—Mr. J. Michell Clarke, in an article on this subject, draws the following conclusions:

1. That the production of intestinal antisepsis is a rational mode of treament of enteric fever, and that beta-napthol is a safe and tolerably efficient agent for this end.

2. That the duration of the disease was shortened in the cases in which he used the drug, and the intensity of the symptoms directly arising from profound disturbance in the alimentary canal was lessened.

3. That the tendency to the occurrence of splenic enlargement, albuminuria, and of secondary complications, such as boils, abscesses, etc., of purulent infective origin, is diminished.

4. That complete convalescence is more speedily and satisfactorily attained, and that there is less risk of a propagation of the disease to others.

In some patients, however, napthol may cause so much gastric disturbance as to prevent its use.—*The Practitioner*, December, 1888.

---

*Action of Inhalations of Ethylene Chloride on the Eye*—M. Panas, after a study of this subject, believes, contrary to the opinion of M. Raphael Dubois: 1. That the corneal trouble caused by inhalations of ethylene chloride are due to a serous infiltration of the corneal parenchyma. 2. That the mechanism of œdema of the corneal tissue depends on the destruction, by the ethylene chloride, of the corneal endothelium, which only protects the cornea against the invasion of the aqueous humor. Panas adds that he has never seen glaucoma after inhalations of chloride of ethylene.—*Progrès Médical*, No. 50, 1888.

*Treatment of Serpiginous Ulcers of the Cornea.*—In the treatment of serpiginous ulcers of the cornea, Dr. Dehenne advises the following course: (1) Open the lachrymal ducts and irrigate the passages with an antiseptic solution (boric acid 4:100). (2) Wash out the conjunctival *cul-de-sac* with sublimate solution, 1:2000. (3) Cauterize the surface of the corneal ulcer by the aid of a fine pointed thermo-cautery at a white heat. (4) If there is pus in the anterior chamber, perforate the cornea with the same point and evacuate the pus. (5) Instil five or six drops of neutral sulphate of eserine solution (1:200) four times daily. —*Union Médical*, January 1, 1889.

---

*Iso-Nitroso-Antipyrin.*—So much has been said lately about the toxic action of mixtures of antipyrin in sweet spirits of nitre, that two observers, Dr. H. C. Wood,[1] of Philadelphia, Pa., and Dr. G. Evans,[1] of Aberdeen, Miss., have made some experiments in this direction, and both claim that ordinary amounts of the mixture are perfectly harmless. The latter observer gave doses of the mixture to animals and man, and found the only effect was that of the antipyrin remaining unchanged. The former experimenter used the isolated product, the iso-nitroso-antipyrin, and found it inert. He says that a very small and harmless amount of cyanogen develops if the mixture stands for some time, particularly on addition of an acid.

---

*Cannabin in Graves' Disease.*—Valieri, after using cannabin in three cases of exophthalmic goitre, recommends the following formulas:—

Cannabin .................. gr. iv ss
Sugar of milk, q. s ......... Mix.
Make 5 pills.
S. To be taken in 24 hours.

Cannabin .................. gr. iv ss
Distilled water ............. ℥ iij
Syrup of orange flowers ..... ℥ j
S. Take in teaspoonful doses in 24 hours .............. Mix.

Or we may prescribe a decoction of 2 or 4 100th parts, or doses of ♏ 15 or 30 of the tincture.—*Wiener med. Presse*, No. 41, 1888.

---

[1] Therapeutic Gazette, Feb. 15, 1889.

